# Navigated direct photocoagulation with a 30-ms short-pulse laser for treating microaneurysms in diabetic macular edema exhibits a high closure rate

**DOI:** 10.1038/s41598-023-33260-6

**Published:** 2023-04-13

**Authors:** Yasuko Ikegami, Tomoyasu Shiraya, Fumiyuki Araki, Takashi Ueta, Taku Toyama, Tomohiko Yanagita, Jiro Numaga, Nobuyuki Shoji, Satoshi Kato

**Affiliations:** 1 Department of Ophthalmology, Graduate School of Medicine, University of Tokyo, 7-3-1 Hongo, Bunkyo-Ku, Tokyo, Japan; 2 Department of Ophthalmology, School of Medicine, Kitasato University, Sagamihara, Kanagawa Japan; 3 Department of Ophthalmology, Tokyo Metropolitan Institute for Geriatrics and Gerontology, Tokyo, Japan

**Keywords:** Outcomes research, Eye manifestations, Imaging and sensing, Eye diseases, Retinal diseases, Diseases

## Abstract

This study carried out direct photocoagulation for treating microaneurysms (MAs) in diabetic macular edema (DME) using a navigation laser system with a 30-ms pulse duration. The MA closure rate after 3 months was investigated using pre and postoperative fluorescein angiography images. MAs primarily inside the edematous area based on optical coherence tomography (OCT) maps were selected for treatment, and leaking MAs (n = 1151) were analyzed in 11 eyes (eight patients). The total MA closure rate was 90.1% (1034/1151), and the mean MA closure rate in each eye was 86.5 ± 8.4%. Mean central retinal thickness (CRT) decreased from 471.9 ± 73.0 μm to 420.0 ± 87.5 μm (*P* = 0.049), and there was a correlation between the MA closure rate and the CRT reduction rate (r = 0.63, *P* = 0.037). There was no difference in the MA closure rate depending on the degree of edema thickness based on a false-color topographic OCT map image. Direct photocoagulation for DME with a short pulse using the navigated photocoagulator resulted in a high MA closure rate in just 3 months and a corresponding improvement in retinal thickness. These findings encourage the use of a new therapeutic approach for DME.

## Introduction

Diabetic macular edema (DME) is the leading cause of visual impairment in patients with diabetes^1^. Microaneurysm (MA) is a contributing factor to the onset of DME^2^ and is a small capillary outpouching resulting from hyperglycemia with focally proliferated endothelial cells and a reduced number of pericytes^3^. Various studies on the numbers, distribution, shapes, and sizes of MAs in diabetic retinopathy^4–7^ have reported that the number of MAs increases with DME progression^8–10^ and that improvement of macular edema is correlated with a decrease in the number of leaking MAs^11,12^.

Direct photocoagulation for MAs (MAPC) remains an important option to treat DME, which results in the cessation of leakage into the retinal space and reconstruction of the disrupted blood-retinal barrier, leading to a reduction in edema^11,13^. Recently, the Navilas® laser system (OD-OS, Teltow, Germany) was developed, which enables high irradiation accuracy along with an automatic eye-tracking delivery system based on a preplanned irradiation design by importing external diagnostic images. This system allows physicians to accurately determine MA in a planned treatment location^14–16^. Using this laser system, a hit rate of 92% was achieved on MAs for DME, which was higher than that of the manual technique (72%)^14^. In addition, the Navilas® system enables us to effectively manage the irradiated area using the digital treatment report, which is useful for evaluating closed MAs where MAPC was performed by comparing pre- and postoperative multimodality images, including fundus photography, optical coherence tomography (OCT), and fluorescein angiography (FA).

Laser photocoagulation has been the only evidence-based treatment for DME since the Early Treatment Diabetic Retinopathy Study (ETDRS) report in 1985^17^, before the advent of anti-vascular endothelial growth factor (VEGF) agents. In general, the pulse duration of laser irradiation in MAPC is 100 ms in actual clinical practice^18–20^. The MA closure rate with conventional parameters (100 ms) using the Navilas® system was 72.4% at 3 months, which was similar to that of the standard manual laser technique^21^. As for the pulse duration, a patterned scanning laser with a short-pulse irradiation time (20–30 ms) was reported in 2006 and has become popular as a minimally invasive laser therapy for the ischemic retina^22^. As tissue invasion depends on the pulse duration, the short-pulse method causes lesser thermal diffusion^23,24^ and retinal damage^25–27^. Suitable MAPC can achieve the closure of MAs, keeping retinal cells intact; however, excessive thermal burns sometimes result in the destruction of the photoreceptor cells and retinal pigment epithelium (RPE), leading to vision loss^28^. Consequently, using MAPC with a short pulse, which is significantly less invasive than the conventional pulse duration (100 ms), is particularly advantageous for treating the delicate macular retina.

Recent evidence shows that VEGF is involved in DME pathogenesis. However, although anti-VEGF injections have been widely used for DME therapy, cases of anti-VEGF–resistant DME have been reported^29^. Focal laser is an important alternative therapy to treat such refractory cases. Therefore, it is significant to investigate the reaction and effect of short-pulse MAPC, which is less invasive, for the future development of treatment. In this study, we performed MAPC in DME with a short-pulse irradiation of 30 ms using the Navilas® system and investigated the rate of MA closure by correlating the pre- and postoperative FA images with the morphological changes detected.

## Results

**Figure 1:**
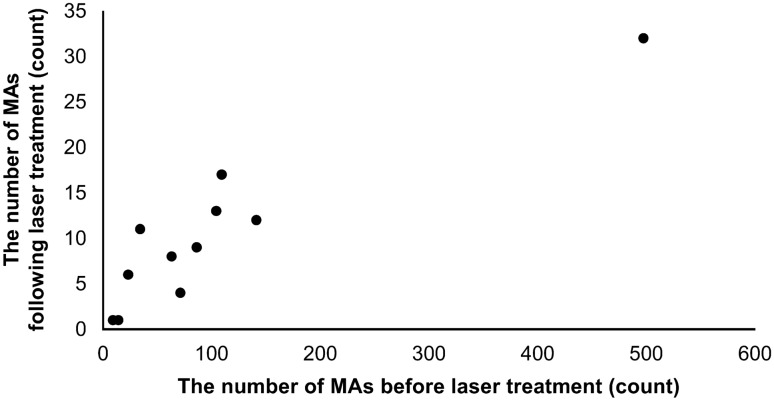
Microaneurysm counts following laser photocoagulation in all cases. MA, microaneurysm.

**Figure 2:**
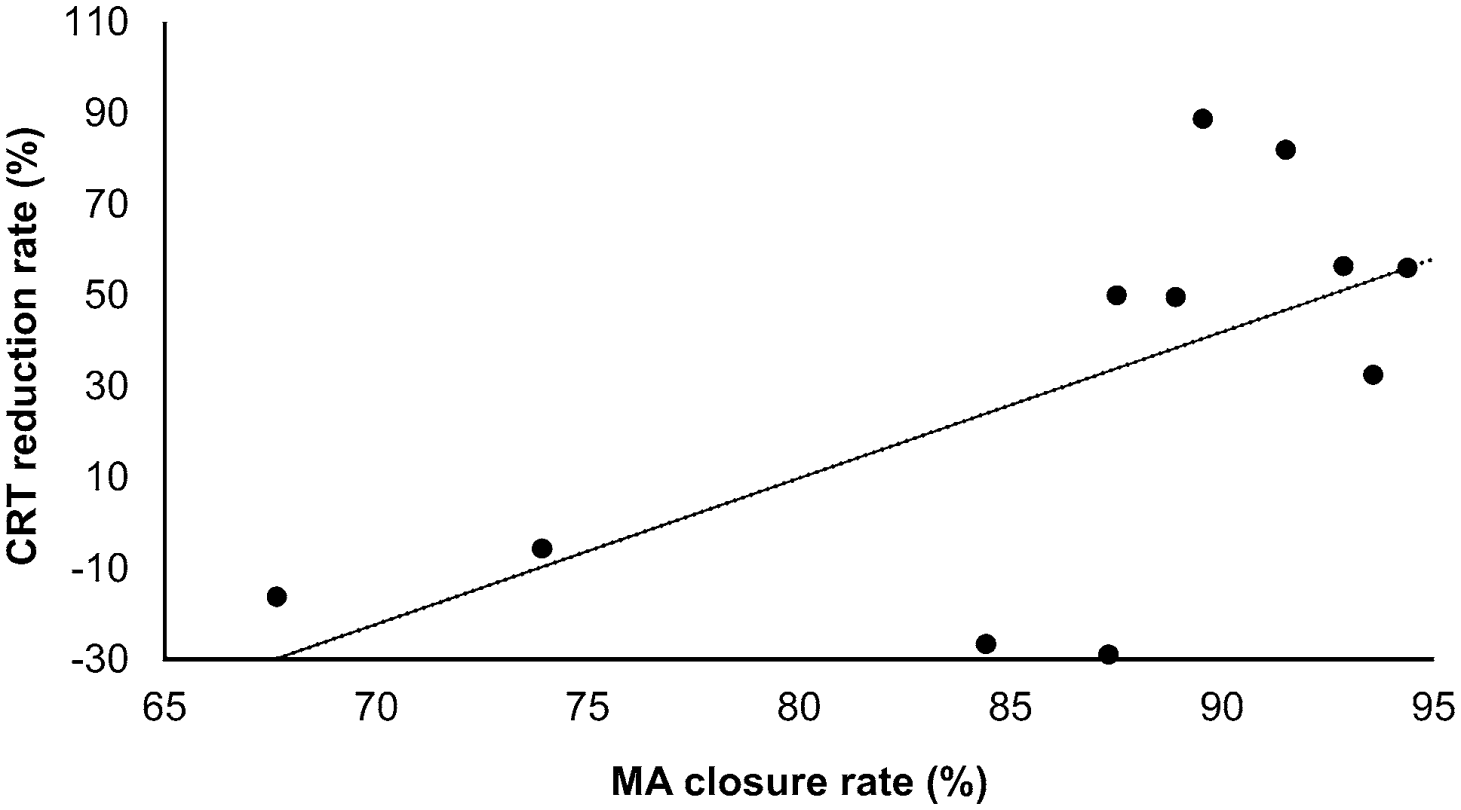
Correlation between microaneurysm closure rate and the central retinal thickness reduction rate. CRT, central retinal thickness.

Leaking MAs (n = 1151) were analyzed in 11 eyes of 8 patients 3 months after they underwent navigated MAPC with a 30-ms pulse duration; Table 1 presents the characteristics of the enrolled patients. The total MA closure rate was 90.1% (1034/1151), and the mean MA closure rate in each eye was 86.5 ± 8.4% (range: 67.7–94.4%). The MA counts following laser photocoagulation in all cases are shown in Fig. 1. There was a correlation between the MA closure rate and central retinal thickness (CRT) reduction rate (r = 0.63, *P* = 0.037) (Fig. 2). However, the correlation between the baseline retinal thickness of the edematous area and MA closure rate in the corresponding sector based on the OCT map was not significant (r = 0.10, *P* = 0.45). We compared the MA closure rate depending on the degree of edema thickness based on a false-color topographic map image, and there was no difference in the MA closure rate, whether the thickness was high (approximately > 500 μm), moderate (approximately 380–500 μm), or outside these edematous ranges (*P* = 0.75, Table 2).

**Table 1:** Baseline characteristics of patients.

Number of eyes (patients)	11 (8)
Male sex, n (%)	7 (64)
Age (years)	67.0 ± 10.2
Diabetes mellitus type, type 2, n (%)	6 (55)
Insulin treatment, n (%)	8 (73)
Hemoglobin A1c (%)	8.05 ± 1.07
Stage of diabetic retinopathy, n (%)	
Mild NPDR	2 (18)
Severe NPDR	8 (72)
PDR	1 (9)
Duration of diabetes (years)	8.2 ± 7.2
Duration of macular edema (years)	3.6 ± 2.8
BCVA (logMAR)	0.53 ± 0.32
CRT (µm)	435.6 ± 98.9
Pseudophakic, n (%)	2 (18)
History of panretinal photocoagulation, n (%)	5 (45)
History of focal photocoagulation, n (%)	4 (36)
Treatment-naive for DME, n (%)	4 (36)

Values are presented as means ± standard deviations. NPDR, non-proliferative diabetic retinopathy; PDR, proliferative diabetic retinopathy; BCVA, best corrected visual acuity; CRT, central retinal thickness; DME, diabetic macular edema.

**Table 2:** Microaneurysm counts and the closure rate depending on the degree of edema thickness according to classification based on a false-color topographic optical coherence tomography map image.

Classification based on a false-color topographic OCT map image	Total MA counts before laser treatment	Total MA counts after laser treatment	Mean MA closure rate (%)
White area (retinal thickness > 500 μm)	634	50	89.9 ± 6.0
Red area (retinal thickness 380–500 μm)	444	60	86.6 ± 10.3
Outside edematous range	35	4	88.2 ± 11.0

n = 11. Values are presented as means ± standard deviations. OCT, optical coherence tomography; MA, microaneurysm.

The mean CRT decreased from 471.9 ± 73.0 μm to 420.0 ± 87.5 μm (*P* = 0.049), and the mean retinal thickness in the edematous area also decreased from 437.5 ± 65.8 μm to 419.1 ± 58.2 μm at 3 months postoperatively (*P* = 0.048). The mean best-corrected visual acuity (BCVA) at baseline was 0.60 ± 0.38 and 0.57 ± 0.37 at 3 months with no significant change over time (*P* = 0.48).

## Discussion

In this study, we performed navigated MAPC with a short pulse duration of 30 ms in DME and investigated the MA closure rate at 3 months after treatment based on FA assessment. The total MA closure rate was 90.1%, and the mean MA closure rate in each eye was 86.5 ± 8.4%. There was a significant relationship between the MA closure rate and the CRT reduction rate. This study also showed no change in MA closure rate irrespective of the degree of edema thickness based on false-color topographic OCT map images.

Laser power, spot size, and pulse duration of irradiation individually affect the morphology of coagulated lesion differently^30^. In the MAPC procedure, the goal is to close the MAs and stop the leakage; however, laser treatment can cause RPE and photoreceptor cell damage^28^. In this regard, MAPC using a short-pulse laser is considered less invasive than the conventional method with a pulsed duration of 100 ms. A previous study that performed navigated MAPC with a pulse duration of 100 ms reported a 72.4% MA closure rate based on FA; however, the closure rate increased over time, reaching 84.6% at 12 months^21^. The short pulse duration of irradiation (30 ms) used in this study resulted in an earlier and higher closure rate of 90.1% at 3 months postoperatively.

One of the reasons for this higher closure rate maybe associated with the fact that a higher laser power is required for short-pulse duration laser therapy as compared to the conventional laser therapy, although lesser thermal diffusion is expected with short-pulse laser. That is, it is possible to exert a localized thermal impact on the target of the irradiated tissue^23,30^. We assumed that a relatively high-power setting was required to induce visible coagulative change in MA; however, the MA itself was instantly coagulated by this high laser power, which may contribute to the high MA closure rate in a relatively short time.

In vitro experiments with cultured RPE cells showed that laser treatment with energy that elicits visible coagulation spots increase pro-apoptotic gene expression^31^. As previously reported, in MAPC with a 100-ms pulse duration, the excessive irradiation energy may have caused thermal damage to the RPE, resulting in the expression of apoptosis-related cytokines,which in turn may have induced MA-closures through their molecular effects over time. It can also be hypothesized that following the incomplete MA closure by conventional photocoagulation, the irregular hyperreflective spots were evaluated as residual MAs in the early postoperative FA^21^. However, apoptosis led to the death of endothelial cells, constituting the MA. This endothelial cell loss associated with MAs, resulted in completely closed MAs overtime, as indicated by the lack of hyperfluorescence in follow-up FA.

In this study, we performed MAPC with short pulses to reduce excessive thermal diffusion into the RPE and outer retinal layers, which suggests that MAs can be closed even with short pulses by accurate irradiation using the Navilas system. In addition, the results show that the rate of decrease in CRT correlated with the MA closure rate, thus reiterating that focal photocoagulation targeting MAs is a strong tool for treating DME.

The localized MAs leaking within the thickened retinal areas significantly contribute to the formation of focal edema^32^. In addition, it should be noted that the thickness of the retinal edema was not uniform. To determine whether the degree of retinal thickness in this edematous area affected the MA closure rate, we categorized this thickness based on a false-color topographic OCT map. However, there was no difference in the MA closure rate between the groups. The present findings suggest that MAPC with a pulse duration of 30 ms using the Navilas® system can produce a stable MA closure effect, regardless of the degree of retinal edema height.

This study was limited by the small number of participants, short follow-up period, and lack of a control group. Moreover, MAs that resolved due to their natural course, rather than in response to photocoagulation, may have been inadvertently included in our results. As we were unable to account for these natural variations, further studies are needed to confirm these results. Nevertheless, MAPC with a short pulse duration of 30 ms using the Navilas system exhibited a high MA closure rate at 3 months postoperatively along with a corresponding improvement in retinal thickness. These findings encourage the use of a new therapeutic approach for DME.

## Methods

### Patients and study design

Patients with DME were recruited from the Department of Ophthalmology of the University of Tokyo Hospital between March 2019 and August 2021. This study was approved by the Institutional Ethics Committee of the Graduate School of Medicine and Faculty of Medicine of the University of Tokyo (#11,986). All patients provided written informed consent after receiving an adequate explanation of the study. All research protocols and measurements adhered to the tenets of the Declaration of Helsinki. Each patient was informed of the treatment options, such as anti-VEGF and steroid therapies, and the risks and benefits of laser photocoagulation. Patients who were reluctant to use anti-VEGF agents for economic or psychological reasons, had recurrent DME despite previous treatments, and were eligible for MAPC because of focal/diffuse DME with a leaking MA, were recruited for this study. The major exclusion criteria were previous intraocular surgery within 6 months; history of treatment with intravitreal anti-VEGF agent or triamcinolone acetonide within 6 months; presence of epiretinal membrane or vitreomacular traction syndrome; or the presence of medial opacity, such as severe cataract, corneal opacity, or vitreous hemorrhage.

### Functional and morphological examinations

All patients underwent complete ophthalmic examinations, including BCVA measurement, intraocular pressure measurement, slit-lamp biomicroscopy, and indirect ophthalmoscopy. BCVA was measured using a standard decimal visual acuity chart, with the decimal BCVA being calculated using the logarithm of the minimum angle of resolution (logMAR) scale. Morphological analysis was performed using spectral-domain OCT (Spectralis, Heidelberg Engineering, Heidelberg, Germany). FA was performed using fundus photography (TRC 50DX; Topcon, Tokyo, Japan) before and after MAPC to clarify the leakage of MAs. On FA, MA leakage was defined as a hyperfluorescent spot observed in the early phase with increasing hyperfluorescence in the later phase^33^.

### Evaluating the morphological changes in spectral-domain OCT

A macular raster scan, consisting of 49 B-scans, was performed using automatic real-time tracking. For the analysis of retinal thickness, a false-color topographic map image that displayed thickness values with numerical averages for the nine sectors defined by the ETDRS was utilized. The OCT map images were obtained before treatment and 3 months after photocoagulation and were set up for perfect comparison using the follow-up program.

To evaluate morphological changes, not only CRT but also mean retinal thickness within the edematous area based on the OCT map was calculated. CRT was defined as the average thickness within the central 1 mm-diameter area. The OCT false-color topographic map shows highly thickened areas (approximately > 500 μm) indicated in white and moderately thickened areas (approximately 380–500 μm) indicated in red. Here, more than half of the areas displayed in white or red were defined as the zones of edematous range, and the numerical averages of the thickness values in these zones were defined as the retinal thickness in the edematous area.

### Navigated direct photocoagulation for MAs

For treatment planning, images of the OCT map and FA were captured by the built-in software of the Navilas 577 s system to create multimodality images. The eye-tracking system linked to the laser photocoagulator ensured the accurate delivery of laser irradiation in coordination with patient eye movements. The early-phase FA image was merged with the OCT thickness map to determine the location of the MAs in DME, and the leaking MAs were selected for MAPC. Laser photocoagulation was performed following pupillary dilation and instillation of topical anesthesia using a Volk Area Centralis® contact lens (Volk Optical, Mentor, OH, USA) by a single retina specialist (YI). The laser parameters were as follows: pulse duration, 30 ms; spot size, 100 µm; single spot; and coagulation power, increased until a grayish/whitish lesion was achieved.

### Identification of closed MAs according to FA images

**Figure 3:**
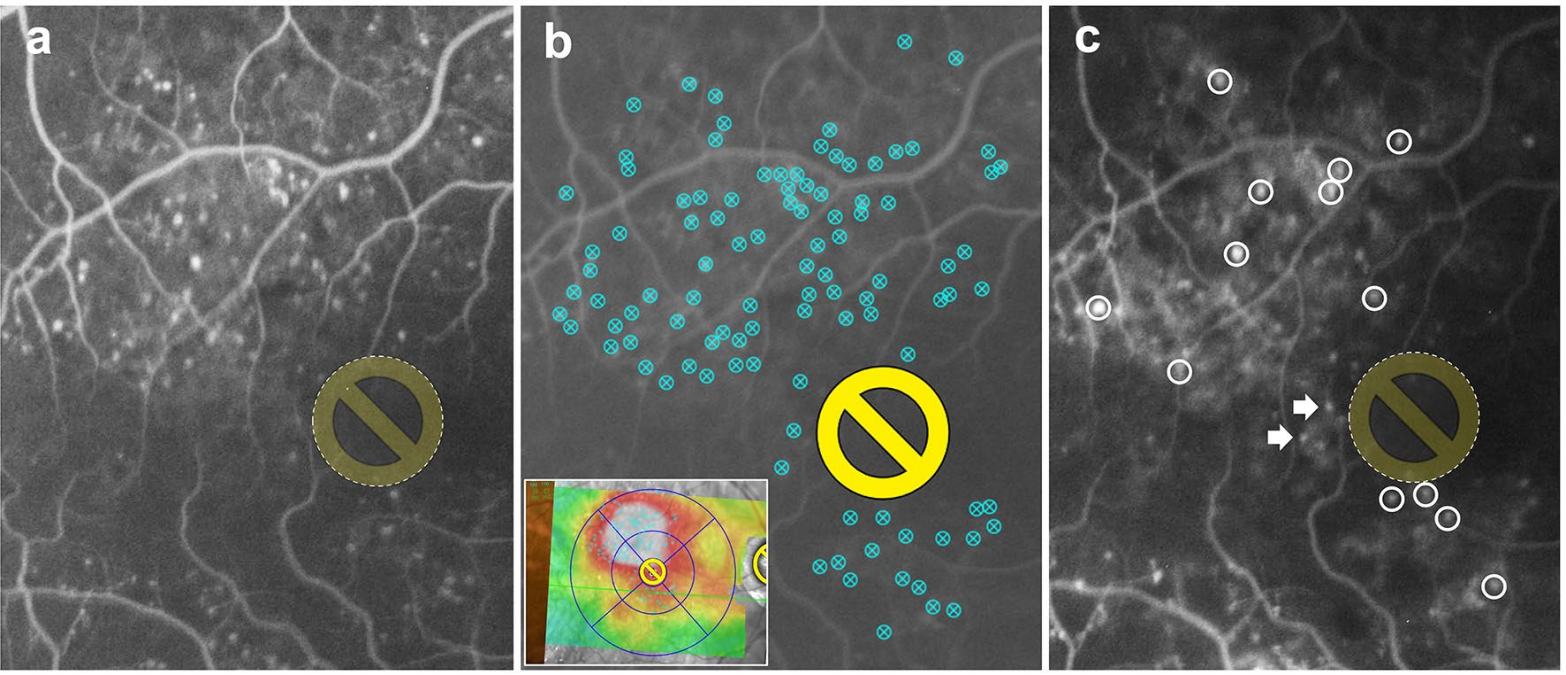
Assessment of microaneurysm closure rate following navigated laser photocoagulation. (**a**) Detection of leaking MA using early-phase FA image before treatment. The yellow circle is an image of the irradiation-prohibited area of the macula. (**b**) Marked laser spots for leaking MA within the edematous area on the OCT map in the laser planning procedure. Additional image below shows the OCT map and laser irradiated spots. (**c**) Postoperative FA image. Closed MAs were defined as an absence of or inactive focal hyperfluorescence. White circles indicate hyperreflective spots, which were considered as residual MAs, and white arrows indicate new MAs appearing during the laser procedure, which were not considered as residual MAs. MA, microaneurysm; FA, fluorescein angiography; OCT, optical coherence tomography.

MA closure following navigated laser photocoagulation at 3 months was assessed using marked laser spots placed on the leaking MAs during the laser planning procedure and on postoperative FA images. Closed MAs were defined as the absence of focal hyperfluorescence in both the early and late phases by three undisclosed retina specialists. In addition, other new MAs appearing after treatment were not considered as residual objects (Fig. 3).

### Outcome measures

The MA closure rate following navigated MAPC was evaluated based on the FA images. Thereafter, the correlations between the MA closure rate and CRT reduction rate, as well as between baseline retinal thickness in the localized zones of edematous area and the corresponding MA closure rate based on OCT maps, were examined. In addition, we compared the MA closure rate depending on the degree of edema thickness.

### Statistical analysis

Data are expressed as mean ± standard deviation. Pearson’s correlation coefficient was used to investigate correlations between the MA closure rate and CRT reduction rate, and Spearman’s rank correlation coefficient was used to investigate correlations between the baseline retinal thickness in specific regions of the edematous area and the corresponding MA closure rate based on the OCT map. One-way analysis of variance was used to examine differences in MA closure rate depending on retinal thickness based on a false-color topographic map image. A paired t-test was used to compare the following parameters pre- and post-laser treatment: CRT, mean retinal thickness of the zones in the edematous area, and BCVA. Statistical significance was set at *P* < 0.05. Statistical analyses were performed using EZR (Saitama Medical Center, Jichi Medical University, Saitama, Japan)^34^.

## Data Availability

The datasets generated during and/or analyzed during the current study are available from the corresponding author on reasonable request.
